# Validation of Padova Classification of Post‐Fundoplication Outflow Obstruction on High‐Resolution Manometry in an International Multi‐Center Study

**DOI:** 10.1111/nmo.70196

**Published:** 2025-11-03

**Authors:** Francesca Forattini, Khanh Hoang Nicholas Le, Luca Provenzano, Matteo Santangelo, Giovanni Capovilla, Arianna Vittori, Matteo Pittacolo, Lucia Moletta, Loredana Nicoletti, Michele Valmasoni, Rena Yadlapati, Renato Salvador

**Affiliations:** ^1^ Department of Surgical, Oncological and Gastroenterological Sciences, School of Medicine University of Padua Padua Italy; ^2^ Department of Gastroenterology and Hepatology University of California San Diego San Diego California USA; ^3^ Department of Medicine, Keck School of Medicine University of Southern California Los Angeles California USA

**Keywords:** antireflux surgery, high resolution manometry (HRM), post‐fundoplication outflow obstruction (PFOO)

## Abstract

**Background:**

Dysphagia after laparoscopic Nissen fundoplication (LNF) is common and may be due to post‐fundoplication outflow obstruction (PFOO) as defined by high‐resolution manometry (HRM) using the Padova Classification. This study aimed to compare clinical and HRM parameters between patients with manometric PFOO and those with a functioning, effective fundoplication (FELF), and to evaluate treatment outcomes in PFOO patients.

**Methods:**

This retrospective study from two international centers included patients who underwent LNF and postoperative HRM between January 2000 and January 2025. Patients were categorized into PFOO (manometric PFOO) and FELF (manometric FELF and normal reflux study) groups. HRM parameters including LES basal pressure, integrated relaxation pressure (IRP), and esophageal body function were compared. Postoperative dysphagia was assessed clinically, and treatment outcomes in PFOO patients were evaluated.

**Results:**

Among 106 patients (62 PFOO, 44 FELF), the PFOO group showed significantly higher median LES basal pressure (41.2 vs. 23.7 mmHg, *p* < 0.01), IRP (19.3 vs. 10.3 mmHg, *p* < 0.01), and LES lengths (*p* = 0.01). PFOO patients had an increased incidence of elevated intrabolus pressure and premature swallows (*p* < 0.01). Dysphagia was reported in 89% of PFOO patients versus 5% in FELF (*p* < 0.01). Of symptomatic PFOO patients undergoing retreatment (pneumatic dilation, redo surgery, or both), 89% achieved symptom improvement.

**Conclusions:**

This first study applying the Padova Classification confirms HRM can distinguish obstructive from functional fundoplications post‐LNF, supporting its diagnostic role in managing postoperative dysphagia.

## Graphical abstract

High‐resolution manometry distinguishes obstructive from functional fundoplication using the Padova classification. In this international study, patients with post‐fundoplication outflow obstruction (PFOO) had distinct manometric profiles and improved dysphagia symptoms following targeted treatment, validating the clinical relevance of this novel classification.

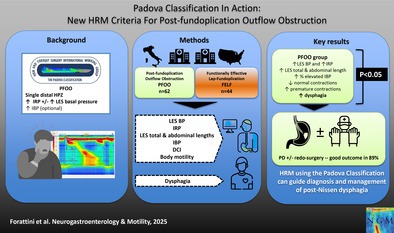

**Summary**

- The Padova Classification helps identify post‐fundoplication outflow obstruction (PFOO) on HRM.
- PFOO patients show higher LES basal pressure and IRP compared with functional fundoplications.
- Dysphagia occurs in 89% of PFOO patients versus 5% in functional fundoplication (FELF).
- Targeted treatments (pneumatic dilation or redo surgery) relieve dysphagia in 89% of cases.
- HRM per Padova criteria supports diagnosis and management of post‐fundoplication dysphagia.

## 1 Background

The standard surgical treatment for GERD is laparoscopic fundoplication (LF), which aims to repair a hiatal hernia, reposition the lower esophageal sphincter (LES) within the abdomen and recreate a flap valve by wrapping the gastric fundus around the LES, completely (Nissen) or partially (Toupet or Dor). LF is a well‐established, safe and effective treatment, achieving symptom relief in up to 90% of patients after 10 years [1, 2, 5]. However, postoperative dysphagia following LF affects up to 12%–25% of patients and typically results from improper wrap positioning, excessive tension during fundoplication construction, or an overly tight hiatoplasty [3, 4, 6]. In clinical practice, a “functioning and effective” fundoplication should exhibit both adequate LES basal pressure and relaxation, sufficient intra‐abdominal LES length, and preservation of normal EGJ morphology (single high‐pressure zone) on postoperative high‐resolution manometry (HRM) [7]. In response to the need for a more comprehensive understanding of postoperative HRM findings, an international multidisciplinary working group recently developed the Padova Consensus [8]. According to this new classification, an excessively tight fundoplication or crural repair can impair the functioning of the “new EGJ” after LF, causing “post‐fundoplication outflow obstruction” (PFOO), a new manometric entity defined by a neo‐LES with increased basal pressure and/or integrated relaxation pressure (IRP), with or without signs of elevated intrabolus pressure (IBP) during swallowing.

This study aims to assess clinical and HRM parameters in patients with a manometric diagnosis of PFOO and secondarily to compare these parameters with those of patients with a functioning and effective LF, using new insights from the Padova Classification.

## 2 Methods

### 2.1 Patient Population

We analyzed and prospectively collected data from all patients treated with laparoscopic Nissen fundoplication (LNF) for GERD, evaluated between 2011 and 2024, with a postoperative HRM at two referral centers for esophageal diseases: the Department of Surgical, Oncological, and Gastroenterological Sciences, University of Padova (Italy), and the Center for Esophageal Diseases, Division of Gastroenterology and Hepatology, University of California San Diego (USA).

### 2.2 Diagnostic Assessment

Patients were evaluated with the Gastroesophageal Reflux Disease Questionnaire (GerdQ) [9], endoscopy and barium swallow to ascertain the presence of anatomical abnormalities. Dysphagia was assessed using a previously described Likert scale that combines severity (0 = none; 2 = mild; 4 = moderate; 6 = severe) and frequency (0 = never; 1 = occasionally; 2 = once a month; 3 = every week; 4 = twice a week; 5 = daily) of dysphagia: a score > 3 was considered positive [7].

After primary LNF, HRM (Manoscan, Medtronic, USA) was performed, according to the standard postoperative protocol [7, 10]. Lower esophageal sphincter (LES) parameters (basal pressure, integrated relaxation pressure, total and abdominal length) and esophageal body function parameters were assessed. The post‐LNF anatomy and physiology were assessed following the Padova classification criteria [8]:

- Functioning and Effective LNF: absence of a separation between crura and neo‐sphincter, with normal LES basal pressure and IRP;
- Disrupted wrap: absence of a separation between high pressure zone (HPZ) and diaphragmatic crura (CD) with low LES basal pressure;
- Intrathoracic fundoplication: separation of the HPZ > 1 cm above the CD;
- Slipped fundoplication: separation of the HPZ > 1 cm below the CD;
- PFOO: absence of a separation between the crura and the neo‐sphincter with elevated IRP (> 19 mmHg) and/or elevated LES basal pressure (> 48 mmHg) with or without elevated intrabolus pressure (IBP).

Esophageal body function was analyzed following the Chicago Classification v4.0. IBP was assessed as a dichotomous variable (normal vs. abnormal) with a threshold of 20 mmHg for supine wet swallows, using the Medtronic system isobaric contour tool [11, 12]. The esophageal acid exposure was assessed using the 24‐h pH‐impedance (Bioview Analysis, Diversatek, USA) or the 96‐h wireless pH monitoring (BRAVO systems, Medtronic, USA). The procedure was performed in all patients at least 15 days after suspending any proton pump inhibitors (PPIs), H2 blockers, or prokinetic agents [13]. An abnormal 24‐h pH monitoring was defined using the Lyon 2.0 criteria [14].

We defined two study groups:

- PFOO group: patients who underwent LNF and whose postoperative manometric tracing met the diagnostic criteria for PFOO, regardless of the presence or absence of dysphagia;
- Functionally Effective Laparoscopic Fundoplication (FELF) group: patients treated with LNF at the University of Padova, with manometric diagnosis of a functioning and effective fundoplication [7, 8] along with normal postoperative pH‐monitoring results, to serve as a well‐characterized control group.

Following the HRM evaluation, the PFOO patients were reassessed in clinic, and patients complaining of persistent dysphagia (Dysphagia score > 3) were offered pneumatic dilation, followed by redo surgery if dysphagia persisted.

We excluded patients assessed with conventional manometry after surgery, those with prior upper gastrointestinal surgery for other diseases, and those with radiologic or manometric signs of disrupted wrap, such as intrathoracic or slipped fundoplication. Furthermore, since the majority of patients meeting the manometric criteria for PFOO had undergone Nissen fundoplication, we excluded patients who had a partial fundoplication (Toupet or Dor) to prevent statistical biases.

### 2.3 Re‐Treatment

All PFOO patients with an abnormal dysphagia score were informed about the available therapeutic options (pneumatic dilation or redo‐surgery), together with the risks and benefits of each option.

Endoscopic pneumatic dilation of the cardia was performed according to the established technique [15]. A Rigiflex balloon dilator (Boston Scientific, Massachusetts, USA) was passed over a guidewire positioned in the stomach and was inflated under direct endoscopic visual control. Alternatively, at UCSD, some patients undergoing FLIP panometry (EndoFLIP, Medtronic, Dublin, Ireland) were treated with an EsoFLIP dilator (Medtronic, Dublin, Ireland), which was distended using saline [16].

The technique of redo‐surgery depended on the original approach (open vs. laparoscopic), adhesions and the intraoperative findings (tight wrap or crura repair) [17].

### 2.4 Postoperative Assessment

Patients were assessed in the outpatient clinic at 1 and 6 months after re‐treatment. During each visit, patient symptoms were evaluated using the Gerd‐Q score and dysphagia scoring system. A barium swallow was obtained at 1 month and physiologic studies (HRM and 24‐h pH monitoring) were repeated at 6 months.

### 2.5 Statistical Analysis

Numerical data were summarized as medians and interquartile ranges, and categorical data as absolute frequencies (*n*) and relative frequencies (%). The study groups were compared by using the Mann–Whitney test for continuous variables, the Chi‐square test and the Fisher test, as appropriate, for the categorical variables. All tests were two‐sided and a *p*‐value less than 0.05 was considered statistically significant. The statistical analysis was carried out with R 4.4 software (R Foundation for Statistical Computing, Vienna, Austria).

## 3 Results

**FIGURE 1:**
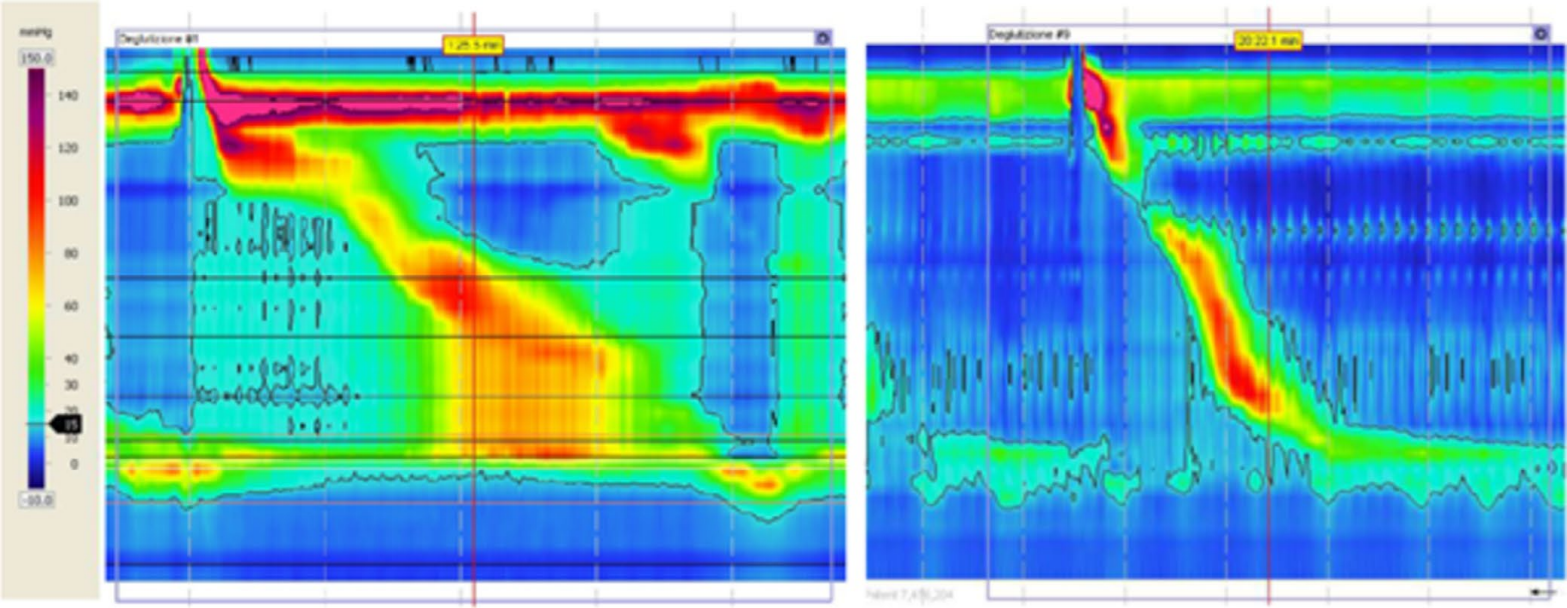
High resolution manometry pathways: PFOO (left) vs. FELF (right).

Of the 263 HRMs reviewed, 157 were excluded. The study included 106 LNF patients: 62 in the PFOO group and 44 in the FELF group (Figure 1). Demographic and clinical parameters are summarized in Table 1. Median patient age was 48 years (IQR 41–58 years), with no difference between groups (*p* = 0.32). Male gender was more prevalent in the FELF group (58% vs. 80%, *p* = 0.02). At primary LNF, two patients had a hiatoplasty mesh reinforcement (one absorbable and one nonabsorbable); both patients belonged to the PFOO group. Among the 62 patients classified as PFOO, 55 (88.7%) had elevated IRP and 14 (22.6%) had elevated LES basal pressure; 48 patients (77.4%) fulfilled criteria based on IRP alone, 7 (11.3%) on LES basal pressure alone, and 7 (11.3%) on both.

**TABLE 1:** Patients' demographic and clinical parameters.

	PFOO (*n* = 62)	FELF (*n* = 44)	*p*
Age, years^a^	53 (41–59)	48 (41–56)	0.32
Sex, M^b^	36 (58%)	35 (80%)	**0.02**
Dysphagia^b^	55 (89%)	2 (5%)	**< 0.01**
No dysphagia^b^	7 (11%)	42 (95%)	
LES basal pressure, mmHg^a^	41.2 (35.7–51.9)	23.7 (18.6–29.0)	**< 0.01**
IRP, mmHg^a^	19.3 (17.5–22.4)	10.3 (8.0–12.6)	**< 0.01**
LES total length, mm^a^	36 (31–47)	31 (25–38)	**0.01**
LES abdominal length, mm^a^	20 (15–27)	14 (4–24)	**0.01**
DCI, mmHg cm/s^a^	1558 (1006–2362)	1272 (720–1991)	0.20
IBP ≥ n.v.^b^	21 (34%)	5 (11%)	**< 0.01**
Esophageal body contractions			
Normal contractions^b^	460/620 (74.1%)	375/440 (85.2%)	**< 0.01**
Weak swallows^b^	59/620 (9.5%)	34/440 (7.7%)	0.32
Failed swallows^b^	25/620 (4.1%)	14/440 (3.2%)	0.51
Premature swallows^b^	76/620 (12.3%)	17/440 (3.9%)	**< 0.01**
Esophageal dysmotility according to CC v4.0^a^			
IEM	6 (9.7%)	3 (6.8%)	0.73
AC	2 (3.2%)	1 (2.3%)	1.0
DES	14 (22.6%)	3 (6.8%)	**0.02**

*Note:* Data expressed as: ^a^median (IQR); ^b^number (%). Significance of bold values indicate statistically significant results of *p*‐values when *p*<0.05.

Abbreviations: AC, Absent contractility; CC, Chicago Classification; DCI, distal contractile integral; DES, Distal esophageal spasm; FELF, functioning and effective laparoscopic fundoplication; IBP, intrabolus pressure; IEM, Ineffective esophageal motility; IRP, integrated relaxation pressure; LES, lower esophageal sphincter; n.v., normal value; PFOO, post fundoplication outflow obstruction.

In the subset of PFOO patients with available preoperative HRM prior to laparoscopic Nissen fundoplication (*n* = 41), median values were: LES basal pressure 22.6 mmHg (16.0–40.7), IRP 7.6 mmHg (4.4–11.0), LES total length 35.0 mm (26.0–43.5), and LES abdominal length 10.0 mm (0.0–20.4). Only IRP (*p* < 0.01) and LES abdominal length (*p* = 0.035) showed significant postoperative changes.

### 3.1 Dysphagia in PFOO vs. FELF

At clinical evaluation after LNF, 89% of patients (55/62) in the PFOO group had an abnormal dysphagia score. In the FELF group, a significantly lower proportion of patients had an abnormal dysphagia score compared to the PFOO group (5% (2/44); *p* < 0.01).

### 3.2 HRM Parameters in PFOO vs. FELF

The PFOO group had a median LES basal pressure of 41.2 mmHg (IQR 35.7–51.9 mmHg) and a median IRP of 19.3 mmHg (IQR 17.5–22.4 mmHg). Median LES total length and abdominal length were 36.5 mm (IQR 31–47 mm) and 20.5 mm (IQR 15–27 mm), respectively. Compared to the FELF group, the PFOO group showed significantly higher LES basal pressure (*p* < 0.01), IRP (*p* < 0.01), longer LES total length (*p* = 0.01), and longer LES abdominal length (*p* = 0.01). Elevated IBP was present in 21/62 (34%) of PFOO patients and in 5/44 (11%) of FELF patients (*p* < 0.01). In the PFOO and the FELF groups, the median distal contractile integral (DCI) was 1558 mmHg‐s‐cm (IQR 1006–2362 mmHg‐s‐cm) and 1272 mmHg‐s‐cm (IQR 720–1991 mmHg‐s‐cm), respectively (*p* = 0.2). Esophageal body contractions were analyzed in the two study groups: compared to the FELF group, the PFOO group had a lower percentage of normal contractions (85.2% vs. 74.1%; *p* < 0.01), and a higher percentage of premature swallows (3.9% vs. 12.3%; *p* < 0.01). Analyzing esophageal peristalsis according to CC 4.0 metrics, there was no statistically significant difference in the prevalence of ineffective esophageal motility (IEM) and absent contractility (AC) between the PFOO and FELF groups: 6/62 (9.7%) vs. 3/44 (6.8%; *p* = 0.73) and 2/62 (3.2%) vs. 1/44 (2.3%; *p* = 1.0), respectively. In contrast, the prevalence of distal esophageal spasm (DES) was significantly higher in the PFOO group (14/62, 22.6%) compared to the FELF group (3/44, 6.8%; *p* = 0.018).

Among the PFOO group, we compared the manometric parameters of patients with and without dysphagia (Table 2). Normal contractions were significantly more frequent in patients without dysphagia compared to those with dysphagia (88.6% vs. 72.4%; *p* < 0.01). Concerning the LES parameters, the IRP was significantly lower in the subgroup without dysphagia compared to those with dysphagia (18.3 mmHg (IQR 16.4–19 mmHg) vs. 19.7 mmHg (IQR 17.8–22 mmHg); *p* < 0.01).

**TABLE 2:** Manometric parameters of PFOO patients with and without dysphagia.

	PFOO no dysphagia (*n* = 7)	PFOO dysphagia (*n* = 55)	*p*
LES basal pressure, mmHg^a^	36.3 (33.1–48.8)	41.6 (35.8–53.2)	0.49
IRP, mmHg^a^	18.3 (16.4–19)	19.7 (17.8–22)	**0.03**
LES total length, mm^a^	34 (32–47)	37 (30–47)	0.84
LES abdominal length, mm^a^	21 (15–23)	20 (14–29)	0.71
DCI, mmHg cm/s^a^	1935 (622–2625)	1496 (1016–2272)	0.98
IBP ≥ n.v.^b^	2 (29%)	19 (35%)	0.68
Esophageal body contractions			
Normal contractions^b^	62/70 (88.6%)	398/550 (72.4%)	**< 0.01**
Weak swallows^b^	3/70 (4.3%)	62/550 (11.3%)	0.09
Failed swallows^b^	1/70 (1.4%)	25/550 (4.5%)	0.35
Premature swallows^b^	4/70 (5.7%)	65/550 (11.8%)	0.16

*Note:* Data expressed as: ^a^median (IQR); ^b^number (%).

Abbreviations: DCI, distal contractile integral; IBP, intrabolus pressure; IRP, integrated relaxation pressure; LES, lower esophageal sphincter; n.v., normal value; PFOO, post fundoplication outflow obstruction.

### 3.3 HRM Parameters in Dysphagic and Non Dysphagic Patients

A supplementary analysis was performed according to the presence of dysphagia (Table S1). Patients with dysphagia (*n* = 57) were older and more frequently female compared to those without dysphagia. They also exhibited significantly higher LES basal pressure and IRP values, as well as longer LES total and abdominal length. Elevated IBP was present in 36% of dysphagic patients versus 12% of those without dysphagia. Regarding esophageal body function, patients with dysphagia showed a lower proportion of normal contractions (71.9% vs. 86.7%; *p* < 0.01) and a higher rate of premature swallows (12.6% vs. 4.3%), with the latter showing a trend towards statistical significance (*p* = 0.06). Finally, DES and IEM were not more frequent among dysphagic patients (22.8% vs. 8.2%; *p* = 0.06 and 12.3% vs. 4.1%; *p* = 0.17, respectively), although DES approached statistical significance. In multivariate logistic regression including variables significant at univariate analysis, IRP was the only independent predictor of dysphagia (OR 1.82, 95% CI 1.35–2.47; *p* < 0.01).

### 3.4 Treatment Outcomes

**FIGURE 2:**
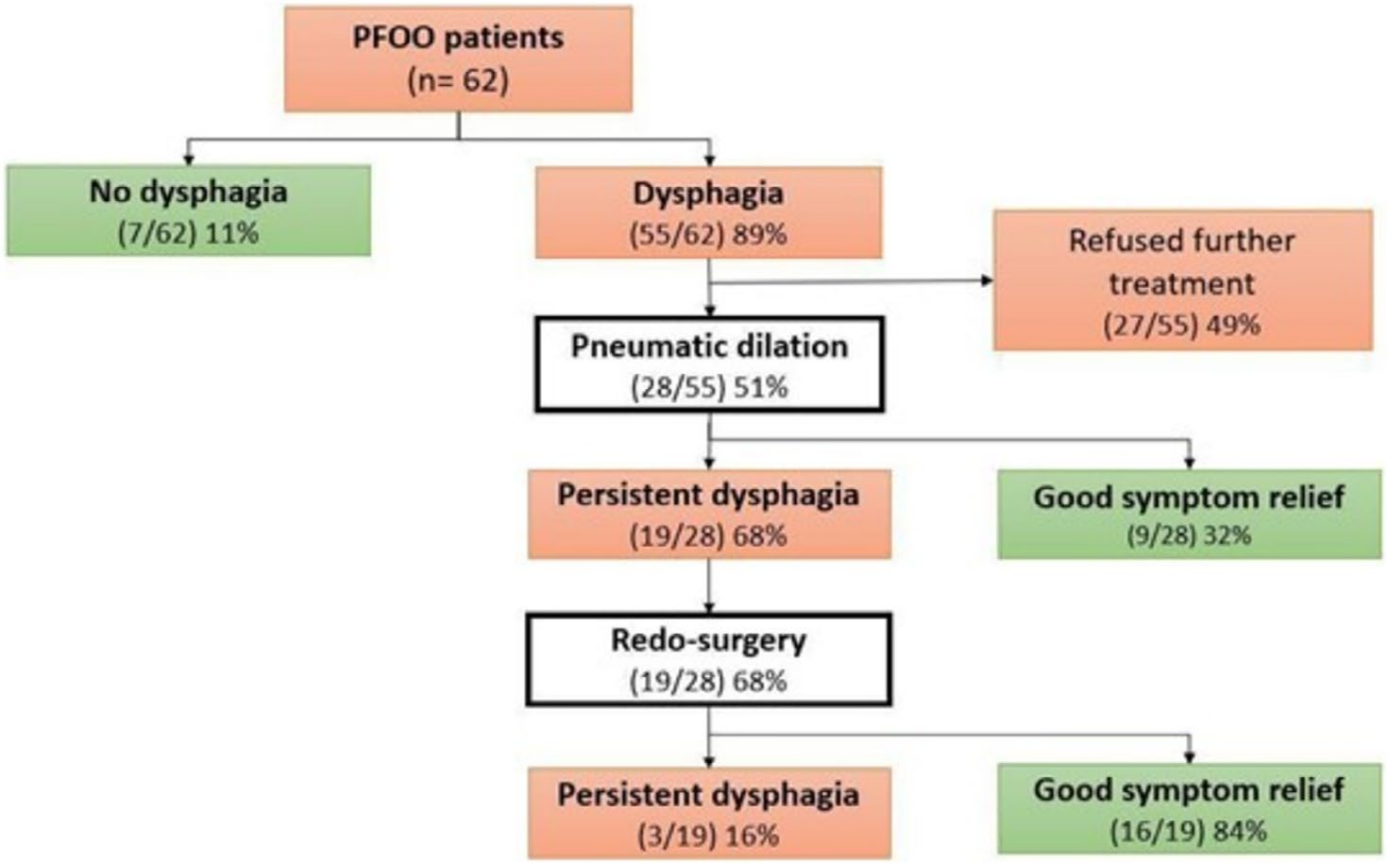
Flow chart of PFOO patients management.

Among symptomatic PFOO patients, 51% (28/55) accepted further treatments. All 28 underwent endoscopic pneumatic dilation with 9/28 (32%) experiencing improvement of dysphagia. The 19/28 (68%) patients in whom PD was not successful in relieving the symptom underwent redo‐surgery: Nissen fundoplication in 2/19 (11%) and Toupet fundoplication in 17/19 (89%). Intraoperative evaluation of these 19 patients identified outflow obstruction attributed to a tight crura in 21% of patients (4/19) and to a tight wrap in 42% of patients (8/19). In seven patients (37%) it was not possible to assess the technical cause of PFOO during surgery. Overall, at a median follow‐up of 126 months (IQR 52–169), PD alone or complementary treatment with PD and redo‐surgery was successful in 25/28 (89%). After persisting failure, one patient underwent esophagectomy due to migration of the nonabsorbable mesh positioned during primary LNF, whereas two patients underwent additional PD (Figure 2). The two FELF patients who reported dysphagia preferred not to receive any additional treatment.

In the retreatment subgroup, no clinical or manometric parameter significantly predicted the success of pneumatic dilatation: the comparison between successfully retreated patients and those with persistent dysphagia did not reveal independent predictors, with only nonsignificant trends for lower IRP and younger age. Complete treatment failure was observed in only three patients, precluding any meaningful statistical analysis.

## 4 Discussion

A major challenge in anti‐reflux surgery remains finding the right balance between antegrade and retrograde bolus flow and optimizing symptomatic control while minimizing postoperative side effects. Structural complications following fundoplication are reported in up to 30% of cases, frequently resulting from technical issues related to the positioning or construction of the wrap [18]. The Padova consensus has recently standardized the interpretation of postoperative HRM findings [8] and provides clear definitions for both a functional and effective LF (FELF) and post‐fundoplication outflow obstruction (PFOO). In our first‐of‐its‐kind multi‐center international study of 106 LNF patients with postoperative HRM we identified three key findings. First, this study validates that the Padova Classification criteria are able to reliably identify patients with postoperative dysphagia due to a tight wrap or tight crura on HRM. Second, we identified that dysphagia symptoms were significantly more prevalent in those with PFOO on HRM (89%) compared to those with a functional and effective LF (5%). Finally, among those with PFOO on HRM, 89% derived symptom relief following a PD +/− redo surgery.

It is well‐established that Nissen fundoplication is associated with higher EGJ resting pressures, higher IRP, and increased esophageal contractility compared to partial fundoplication [7, 19, 20, 21]. It is therefore critical to establish parameters that can effectively distinguish between expected post‐LNF findings on HRM which represent a functional and effective LF versus parameters which represent PFOO. In this study, the PFOO group indeed exhibited significantly higher LES basal pressures, IRP, and greater LES total and abdominal lengths compared to the FELF group. Our results also suggest that the presence of postoperative dysphagia in patients with a manometric diagnosis of PFOO is associated with a higher IRP, while LES basal pressure and intrabolus pressure are not discriminatory. Although statistically significant, the small difference in the median IRP (1.4 mmHg) is unlikely to be clinically relevant. Rather, our findings suggest that preserved contractile activity may act as a compensatory mechanism, as supported by the higher proportion of normal contractions in non‐dysphagic patients. Future studies need to confirm these results, especially for symptomatic patients. Previous meta‐analyses comparing Nissen and Dor/Toupet fundoplication found no significant differences in postoperative heartburn or PPI use, but a higher incidence of postoperative dysphagia after Nissen [22, 23, 24, 25, 26]. Recent guidelines suggest tailoring the choice of surgical technique to patient priorities, balancing symptom control with the minimization of side effects such as dysphagia [27]. Consistent with the existing literature, in our database PFOO was predominantly observed after Nissen fundoplication, leading us to exclude partial fundoplication cases from the analysis. Consequently, our group of comparison consisted of asymptomatic patients with a FELF after Nissen.

Esophageal motility disorders are frequently encountered in the preoperative assessment for antireflux surgery [14, 28], and in the postoperative period it is difficult to distinguish a preexisting dysmotility from a new onset dysmotility. Patients with GERD and esophageal motility disorders are often considered suboptimal candidates for Nissen fundoplication given the increased risk of persistent dysphagia. In our cohort, a higher percentage of premature contractions was found in patients with PFOO. Premature swallows post‐LNF may represent a reactive motility pattern secondary to hiatal flow resistance caused by a tight fundoplication or crural closure. The lack of pre‐LNF HRM data in our study limits the ability to draw conclusions about preexisting versus new onset dysmotility. On the other hand, in our study patients in the FELF group had a higher percentage of peristaltic contractions. A recent single‐center study showed that a functionally and effective fundoplication significantly correlates with the normalization of preoperative esophageal dysmotility, supporting the hypothesis that esophageal peristaltic dysfunction could be a result of reflux exposure and mucosal injury [13]. Our results further support this hypothesis.

In the case of a tight fundoplication and an intact wrap below the diaphragm, endoscopic dilation can be proposed as first‐line treatment. According to case series, endoscopic dilation relieves symptomatic dysphagia in up to two‐thirds of cases [6]. In our cohort, endoscopic treatment with Rigiflex pneumatic dilation or EsoFLIP was accepted by approximately half (51%) of the symptomatic patients, achieving symptom control in 32% of treated cases. Patients who continued to experience dysphagia despite endoscopic intervention were subsequently referred for surgical revision. The standard approach involved takedown of the previous wrap and hiatoplasty, a new hiatal repair and finally a redo fundoplication. Considering that redo surgery was performed due to dysphagia, a partial fundoplication was generally preferred to a complete fundoplication to reduce the possibility of failure. As reported in the literature, redo fundoplication achieves lower success rates compared to primary surgery. According to a meta‐analysis by Schlottmann et al. [29], laparoscopic redo fundoplication resulted in symptom improvement in 78.5% of cases and improved quality of life in 80.6%. In our series, symptom relief was achieved in 84.2% of patients undergoing surgical revision. However, it must be emphasized that surgical revision is technically challenging due to adhesions and distorted anatomy, with an increased risk of complications such as esophageal or gastric perforation and leaks, vagal nerve injury, or bleeding [30, 31].

In patients undergoing retreatment—endoscopic alone or combined with surgical revision—we attempted to differentiate whether the primary cause of dysphagia and delayed esophageal emptying was a tight wrap or a tight crura. In our cohort, 43% of PFOO cases were attributed to a tight wrap, while 28.5% were attributed to a tight crura; however we found no correlation with HRM parameters.

This study is the first of its kind to assess the validity of PFOO HRM parameters per Padova consensus. There are many strengths to this study including the multi‐center, international study design and inclusion of the FELF group serving as a robust comparator to PFOO. Limitations of our study must be acknowledged. First, the relatively small sample size, especially for the subgroups (asymptomatic, not retreated, PD and redo‐surgery), limits our ability to use multivariable analysis. Second, the choice of surgical retreatment technique was influenced by the nature of the primary surgery. Since many of the patients in the PFOO group were treated at other hospitals, manometric data before primary surgery were limited. Furthermore, our study design was retrospective. Future prospective studies are needed to better define the role of HRM in the diagnosis and management of PFOO. Finally, our follow‐up protocol did not include endoscopy, impedance planimetry or barium swallow at the same time as HRM, limiting the ability to correlate manometric findings with anatomical abnormalities.

## 5 Conclusions

This is the first study evaluating the HRM parameters of patients with PFOO after LNF, following the new Padova Classification. This study confirms the ability of HRM to discriminate an obstructive fundoplication from a functional one, and therefore, the diagnostic role of the Padova Classification to assess post‐LNF dysphagia and guide management.

## Author Contribution

**Francesca Forattini:** conceptualization; study design; data collection; data interpretation; formal analysis; manuscript drafting; manuscript revision. **Khanh Hoang Nicholas Le:** data collection; data interpretation; manuscript drafting; manuscript revision. **Luca Provenzano:** study design; data interpretation; manuscript drafting; manuscript revision. **Matteo Santangelo:** data collection; data interpretation; manuscript drafting; manuscript revision. **Giovanni Capovilla:** data interpretation; manuscript drafting; manuscript revision. **Arianna Vittori:** data collection; manuscript drafting; manuscript revision. **Matteo Pittacolo:** data interpretation; manuscript drafting; manuscript revision. **Lucia Moletta:** data interpretation; manuscript drafting; manuscript revision. **Loredana Nicoletti:** data collection; manuscript drafting; manuscript revision. **Michele Valmasoni:** study design; data interpretation; manuscript drafting; manuscript revision. **Rena Yadlapati:** conceptualization; study design; supervision; data interpretation; manuscript drafting; manuscript revision. **Renato Salvador:** conceptualization; study design; supervision; data interpretation; formal analysis; manuscript drafting; manuscript revision. All authors read and approved the final version of the manuscript.

## Disclosure

R.Y. reports consulting relationships with Phathom Pharmaceuticals, StatLinkMD, Braintree Pharmaceuticals, Reckitt Benckiser Healthcare Ltd., and Medtronic. She serves on the advisory board of RJS Mediagnostix. All other authors declare no conflicts of interest related to this manuscript.

## Conflicts of Interest

The authors declare no conflicts of interest.

## Supporting information


**Table S1:** Patients' demographic and clinical parameters according to presence of dysphagia.

## Data Availability

The data that support the findings of this study are available from the corresponding author upon reasonable request.
