# Supplementary material for: Validation of Padova Classification of Post‐Fundoplication Outflow Obstruction on High‐Resolution Manometry in an International Multi‐Center Study
Source: Neurogastroenterol Motil. 2025 Nov 3;37(12):e70196. doi: 10.1111/nmo.70196 (PMC12623295; doi:10.1111/nmo.70196)
Supplement: Supplementary file 1 — Table S1: Patients' demographic and clinical parameters according to presence of dysphagia. [file NMO-37-e70196-s001.docx]

**Table S1**. Patients’ demographic and clinical parameters according to presence of dysphagia.

|  | **dysphagia**  (n = 57) | **no dysphagia**  (n = 49) | **p-value** |
| --- | --- | --- | --- |
| Age, years * | 55 (42-60) | 47 (41-54) | 0.18 |
| Sex, M ^ | 28 (49%) | 41 (84%) | **<0.01** |
| LES basal pressure, mmHg * | 37.1 (32.0-41.2) | 25.4 (18.9-29.8) | **<0.01** |
| IRP, mmHg * | 19.5 (19.0-20.9) | 11.1 (9.0-13.1) | **<0.01** |
| LES total length, mm * | 37 (30-47) | 32 (27-39) | **0.03** |
| LES abdominal length, mm * | 20 (14-29) | 16 (7-23) | **0.01** |
| DCI, mmHg cm/s * | 1496 (970-2259) | 1358 (800-2017) | 0.37 |
| IBP ≥ n.v. ^ | 20 (36%) | 6 (12%) | **<0.01** |
| Esophageal body contractions |  |  |  |
| Normal contractions ^ | 410/570 (71.9 %) | 425/490 (86.7 %) | **<0.01** |
| Weak swallows ^ | 62/570 (10.9 %) | 31/490 (6.3 %) | 0.40 |
| Failed swallows ^ | 26/570 (4.6 %) | 13/490 (2.7 %) | 0.12 |
| Premature swallows ^ | 72/570 (12.6 %) | 21/490 (4.3 %) | 0.06 |
| Esophageal dysmotility according to CC v4.0 *  IEM  AC  DES | 7 (12.3%)  2 (3.5%)  13 (22.8%) | 2 (4.1%)  1 (2%)  4 (8.2%) | 0.17  1.0  0.06 |

Data expressed as: * median (IQR); ^ number (%)

Abbreviations: PFOO: *post fundoplication outflow obstruction*; FELF: *functioning and effective laparoscopic fundoplication*; LES: *lower esophageal sphincter*; IRP: *integrated relaxation pressure*; DCI: *distal contractile integral;* IBP: *intrabolus pressure;* n.v. *normal value; CC: Chicago Classification; IEM: Ineffective esophageal motility; AC: Absent contractility; DES: Distal esophageal spasm.*
